# Assessing Canadians Health Activity and Nutritional Habits Through Social Media

**DOI:** 10.3389/fpubh.2019.00400

**Published:** 2020-01-14

**Authors:** Neel Shah, Gautam Srivastava, David W. Savage, Vijay Mago

**Affiliations:** ^1^Department of Computer Science, Lakehead University, Thunder Bay, ON, Canada; ^2^Department of Mathematics and Computer Science, Brandon University, Brandon, MB, Canada; ^3^Research Center for Interneural Computing, China Medical University, Taiwan, China; ^4^Northern Ontario School of Medicine, Thunder Bay, ON, Canada

**Keywords:** data analysis, natural language processing, social media analysis, health analysis, machine learning, calories and physical activity

## Abstract

When conducting data analysis in the twenty-first century, social media is crucial to the analysis due to the ability to provide information on a variety of topics such as health, food, feedback on products, and many others. Presently, users utilize social media to share their daily lifestyles. For example, travel locations, exercises, and food are common subjects of social media posts. By analyzing such information collected from users, health of the general population can be gauged. This analysis can become an integral part of federal efforts to study the health of a nation's people on a large scale. In this paper, we focus on such efforts from a Canadian lens. Public health is becoming a primary concern for many governments around the world. It is believed that it is necessary to analyze the current scenario within a given population before creating any new policies. Traditionally, governments use a variety of ways to gauge the flavor for any new policy including door to door surveys, a national level census, or hospital information to decide health policies. This information is limited and sometimes takes a long time to collect and analyze sufficiently enough to aid in decision making. In this paper, our approach is to solve such problems through the advancement of natural language processing algorithms and large scale data analysis. Our in-depth results show that the proposed method provides a viable solution in less time with the same accuracy when compared to traditional methods.

## 1. Introduction

Every year, Internet access is multiplying at a rate of 7% around the world (1). The level of yearly growth of social media users in Canada however is almost twice as high at 13%. Canada is a good representation for Internet usage with regards to the rest of the world as Canada had a 36.79 million people as of 2018, and among them, 33.05 million are Internet users. This is almost 90% of total population (1). As Internet access and quality increases, it creates an ideal condition for the growth of social media and other online activities. From 2017 to 2018 alone, Canadian's social media penetration reached 68% of the total population with 25.56 million people. The reason behind the exponential growth of social media users is mainly due to the technological advancement of smartphones and qualitative Internet services (with an average speed of Internet 45.64 Mbps in Canada) (1). This shows how deeply social media and the Internet has penetrated Canadian society. An average Canadian spends approximately 6 h of their time every day on the Internet. Eighty-nine percent of the total population use the Internet daily for various activities (1). Smartphones are essential for social media, as they enable users to share their activities with ease of accessibility when compared to traditional social media devices such as computers. In current applications we see cameras integrated right into the application to upload content instantly without the hassle of conventional equipment. In Canada, smartphone users are growing with a rate of 6% every year which will increase the usage of social media, the Internet and different online services (1). This naturally generates an enormous amount of data and information which can be cultivated to form trends. Twitter has the most significant amount of activity among the social media platforms, at 7.2 million monthly active users all over Canada. The raw data collected includes all types of information from reviews of restaurants/products, political views, user's likes/dislikes, daily routines, just to name a few. Since Twitter provides a qualitative source of information that is a good measure of all social media platforms, in this paper our approach is to consider Twitter for analysis of public health as was originally shown in Dodds et al. (2). There are many factors which effect the quality of life and are complicated to measure directly. Presently, the best technique used to measure the quality of life are traditional surveys (3). However, the problem with these techniques include the types of data collected, actual collection of data, cost, degree of randomness, and time involved with the survey. These are all mitigating factors. Due to this conventional method, the chances of error are also increased. This in turn effects the decision of health policies and monitoring as it is not a proper representation but only a skim of the actual state of health within a given demographic area.

By studying the health of the population, trends can be formed with regards to prevalent health conditions. For example, diabetes, cancer, and heart conditions (4). Many of these health conditions are correlated with nutrition and level of daily physical activity. Health policy creators within government know this and conducts surveys, and programs to analyze the current health of nations (5). They can then use the information collected to put in appropriate policies and programs in order to help the population stay healthy and active.

The rest of the paper is organized as follows. In section 1, an introduction was given. Next, related works are discussed to get an idea of the similar current research being done in our field of health analysis in section 2. We then discuss the limitations in data analysis and how they can be solved in section 2.1. Our methodology is presented which includes: data cleaning, creating a database, phrase detection, and model training in section 3. Final results are given and form a detailed health analysis for Canada in section 4. Finally, this work concludes by summarizing our results and discuss the future possibilities in section 5.

## 2. Related Work

Google Flu Trends was a real-time flu detection tool based on Google search query (6). If individuals search for a solution for the flu or any medical information related to the flu, the algorithm uses that information and considers their location as a potential flu affected area (6). However, the algorithm was proven to be ineffective. Paul and Drendze (7) gave a correlation when comparing cancer tweets, showing that there are higher obesity and tweets regarding smoking. They also found a negative relationship between health care coverage and tweets posted about diseases. With more sophisticated algorithms the accuracy of the data increases and this can be used to discover more true trends when looking at Twitter for health analysis.

Shawndra et al. (8) found that people who search about sodium content per recipe directly correlated with the number of people admitted in the emergency room of a major urban Washington hospital for congestive heart failure. Eichstaedt found that sentiment analysis of tweet language outperforms the traditional socioeconomic surveys for predicting heart disease at the country level (9). They correlated the growth of negative emotions in Twitter with the risk factor of heart disease on a large scale. This shows that social media analysis can be more effective than traditional surveys and may be the next step of methodology for future analysis done by the government.

Culotta et al. (10) analyzed tweets which contain the daily habits of the account holders. The results were a “deep representation” of the US community in regards to their daily negative engagement concerning their routine such as watching television, playing, or reading. Abbar also did an analysis of data on Twitter for caloric analysis at the country level. They classified food-related tweets and found the caloric value of such food. This analysis gave a brief understanding of the food habits of the people in different demographic areas (11). Subsequently, Lexicocaloricmeter (LCM) became one of the most sophisticated approaches toward the health analysis of people at the country level. This is done by utilizing social media. LCM is an online instrument that is designed for measuring social, physical, and psychological examination at a large scale. Sharon et al. (12) developed it for public health monitoring and to create health policies through data-centric comparison of communities at all scales. Oversimplification exists in data analysis which means that the data is being classified in basic categories. Doing this results in looking only at the data present instead of looking deeper into the meaning or relevance of the data. This methodology is known to cause bias. An example of this is a piece of data from a Twitter account that says “**the test was a piece of cake**.” This idiomatic expression that has very little to do with food. Instruments like LCM will take this data as a food tweet and add it to is trends. This causes errors and inaccurate trends which needs to be addressed in future models. Models need to have a resistance to oversimplification. LCM extracts text related to caloric input and caloric output and calculates their caloric content (13, 14). They also use food phrases from a 450-plus database and physical activity phrases from a 550-plus database. The second step is to group categorically similar words and phrases into small pieces called lemmas. They then assign caloric values to it, based on the food and physical activity. To get these lemmas, they use a greedy selection algorithm. Food caloric value is represented as *C*_*in*_ and activity caloric value is represented as *C*_*out*_. *C*_*rat*_ is calculated as shown in Equation (1).

(1) $$\begin{array}{l} C_{rat}=\frac{C_{in}}{C_{out}} \end{array}$$

To find the average caloric value of different provinces or countries, frequency of all food and activity related words is counted and then caloric values to all words are assigned. Next, the standard *C*_*rat*_ formula is used to compute the caloric ratio of each place. The authors consider 80.7 kilograms as the average weight for metabolism equivalent of tasks; this is subtracted from the calorie's physical activity value.

### 2.1. Limitations

For simplicity, the LCM did not use any filter for tweets beyond their geographic locations. This causes bias in the data-set because the user may live/eat in different locations. This also causes the users eating habits to affect another location's data-set instead of affecting there home location data-set. For example a given user might be from Toronto and go on a trip and eat in Montreal. With LCM's current filter the user's data will affect the data-set gathered from two separate locations instead just Toronto as it should. This causes a loophole in the data-set that will cause inaccuracy.

LCM's data-set is quite limited with only 451 food phrases (15). The food phrase data-set has the most common food names which limits its applicability. Also when people talk about the food, it can be called anything such as the name of special dish in a certain restaurant (15). Different cultures have different foods and this is very important in a country as diverse as Canada. So the database of food phrases in our model must be large in order to accommodate for all possibilities in order to be accurate.

Another limitation of LCM is that the Twitter account may talk about food or an activity in a metaphorical perspective. Food words are commonly used in idiomatic expressions in the English language. Some examples include: “**bring home the bacon**,” “**crying over spilled milk**,” and “cup of tea.” LCM will still consider these phrases as food items in their system and assign values to them. The approach used in LCM cannot solve this problem, and therefore creates bias in the system. An example of this includes if a person tweets the phrase “**you are the apple of my eye**,” the present algorithm will consider apple as a food. But in this case, it is not related to food. Also, a lack of Natural Language Processing (NLP) understanding of such approach creates higher chances for the bias output (16). Due to this, unnecessary data will enter the data-set and create false trends, over-fitting and decrease accuracy of the overall analysis.

## 3. Methodology

**Figure 1:**
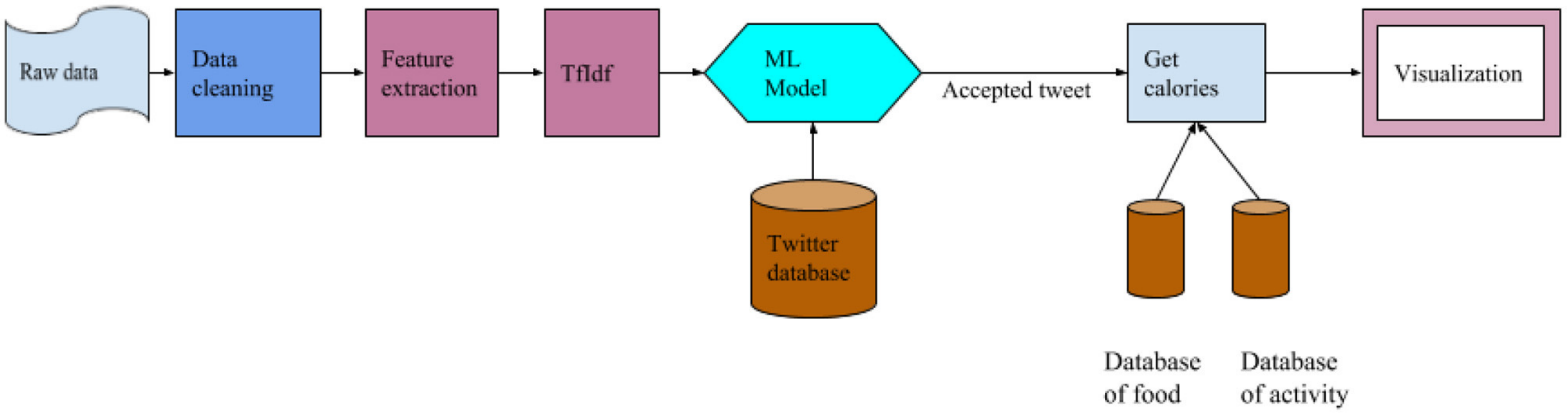
Architect of the analysis system.

In the design of our system, the focus of the system was placed on large scale analysis of social media data with regards to health analysis. The focus of the system is also on training a NLP model based on a large amount of data that is processed to get factual information about the health of Canadians. Figure 1 shows the architecture of our health analysis system. It is divided into two subsections, namely training (offline mode) and the analysis component (active system). As shown in Figure 1, the first step is to collect all the raw data. To manage and process data, Elasticsearch system is used which is designed and developed through Elasticsearch locally at Lakehead University's High-performance computing facility (17). It can handle and analyze Terabytes of text data with a low time overhead. This helped collect the necessary data very efficiently from the pool of data. Once the pool of data was collected, the next step was data cleaning.

### 3.1. Data Cleaning

Data cleaning is crucial when dealing with user's raw data such as tweets, feeds, or chats, as shown in Box 1. Raw data is not structured or cleaned unlike typical formats such as blogs or essays. When tweets are written, it may include hashtags, slang words, emojis, emoticons, and unstructured data. Because of that, raw data is used as a feature in the model as an input; to make this data more sensible and more reliable for the model it must be cleaned.

Box 1 **Data cleaning examples.**

**Step 1**: Emoji or Emoticons gave there respective meaning

**Original text**: “I am getting 2 old to be mango Gonna retire soon and be joesh #ROFL :-)”

**Processed text**: “i am getting 2 old to be mango gonna retire soon and be joesh #ROFL Happy face smiley”

**Step 2**: Covert all text in lower-case characters

**Original text**: “I am getting 2 old to be mango Gonna retire soon and be joesh #ROFL”

**Processed text**: “i am getting 2 old to be mango gonna retire soon and be joesh #rofl happy face smiley”

**Step 3**: Removing stop words

**Original text**: “i am getting 2 old to be mango gonna retire soon and be joesh #rofl”

**Processed text**: “getting 2 old mango gon na retire soon joesh #rofl happy face smiley”

**Step 4**: Removing special characters

**Original text**: “getting 2 old mango gon na retire soon joesh #rofl”

**Processed text**: “getting 2 old mango gon na retire soon joesh rofl happy face smiley”1

**Step 5**: Removing numbers

**Original text**: “getting 2 old mango gon na retire soon joesh rofl”

**Processed text**: “getting old mango gon na retire soon joesh rofl happy face smiley”

In the first step of data cleaning, all Emojis or Emoticons are converted to there respective meaning through the “emot” open source library given in Shah (18). It helps to understand the text when a name that is not in the database. When emojis are related to food, it will be easy to understand that the tweet is related to food. In the next step, all text is converted into lower case which makes word matching and processing easy in further processes. Step 3 is removing stop words which help to eliminate unnecessary features in our model (*the, a, an, when, what*). The next step is to remove special characters. Finally, numbers are removed. This is because numbers are not useful for identifying whether the text is related to food or not and it is also not a source of information for our analysis. Removing all unnecessary text or data will limit the size of the feature matrix and speed up the training and classification task as some features are directly propositional to the speed of model training. As the number of features increases the speed of training the model also increases as given in Batista et al. (19). Here, hashtags are not removed because it gives valuable information. For example, when users talk about specific foods which are not common but use hashtags alongside text, for example #burger#delicious, then the user is talking about a burger or some other food which can be quickly identified. So, special characters were removed while keeping hashtagged text.

### 3.2. Database

To calculate the caloric value, two types of data-sets are needed: first for food and its caloric values and the second for activity and its caloric-burn value. When a data-set is gathered for food, our research found out that there is not even a single data-set available which includes the different types of food items and their nutrition values. At present, the Canadian Food Nutrient Database and USDA Food Composition Databases are the main sources of information related to food and nutrition facts of the food in Canada. But the limitation with these databases is the lack of data in terms of specific foods/items such as “**Chicken masala**” or “**Penne arrabiata**.” Usually, people tweet about specific items they eat during their meal at a restaurant or any other place. It means the present data-set is very domain-oriented for things such as fast food, vegetables, or frozen foods, but they will not contain all the major types of food that people talk about on social media as shown before. There are other problems after getting the data-set. First, to find what type of specific food the users talk about. Next, to find the caloric value of that specific food. To solve these problems, a new data-set is needed that combines different food domains which contain all major foods and their different nutrition values. This is why, “**Food in one**” dataset was created which includes a combination of all open source data-sets such as the Open Food Facts which is a major source of food names, Canadian Nutrient File, and USDA Food Composition Databases. Table 1 shows the structure of current food data-set.

**Table 1:** Food database.

**Name**	**Data**
food_name	Name of the food
food_ingredients	Ingredients use to make the food
fat_100g	Fat per 100 g of food
energy_100g	Energy value per 100 g of the food
carbohydrate_100g	carbohydrate value of that food at 100 g

Our newly created data-set contains 338, 889 foods with all the required information. This is an open source database available at DataLab. This includes all different types of major food sources like fruits, vegetables, fast food, and regular food. In our data-set, more than 70% of the food items are from the USA, Canada, and France. This is because our focus is mainly on Canada's health situations and these are the main sources of food in the Canadian market.

**Figure 2:**
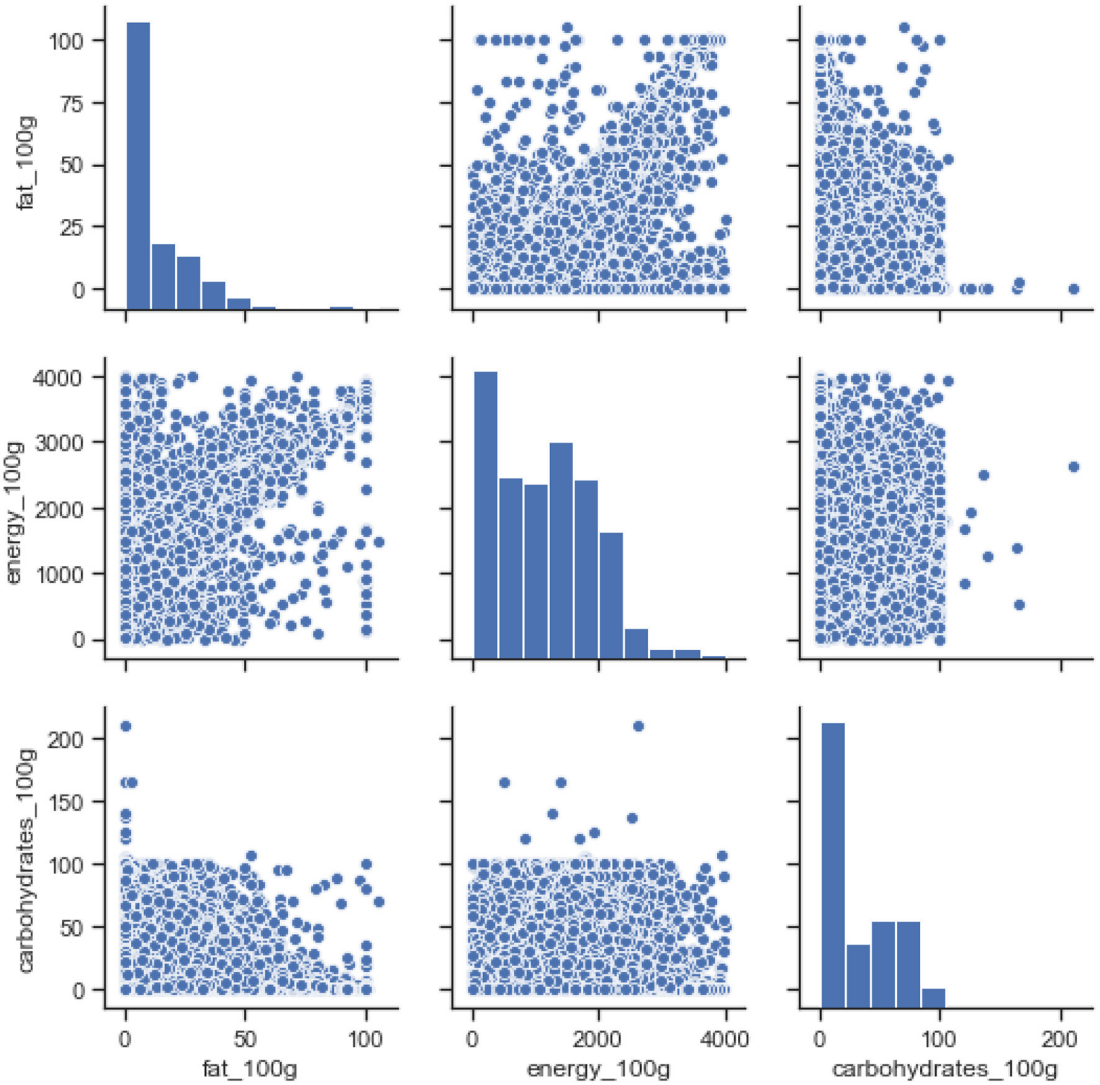
Nutrition value of all food types in the database.

To understand the nutritional value of all food items in the database a Normalized Kernel Density Estimation KDE is used. Figure 2 is the KDE diagram of all the food that is present in the data-set. This is along with their nutrition values including fat, carbohydrate, and energy per 100 grams. As can be seen in the second bar chart energy values mostly lie between mid-range while the fat bar chart has diverse values from an extreme high to an extreme low. This represents the diverse nature of our data-set that includes various type of foods.

**Figure 3:**
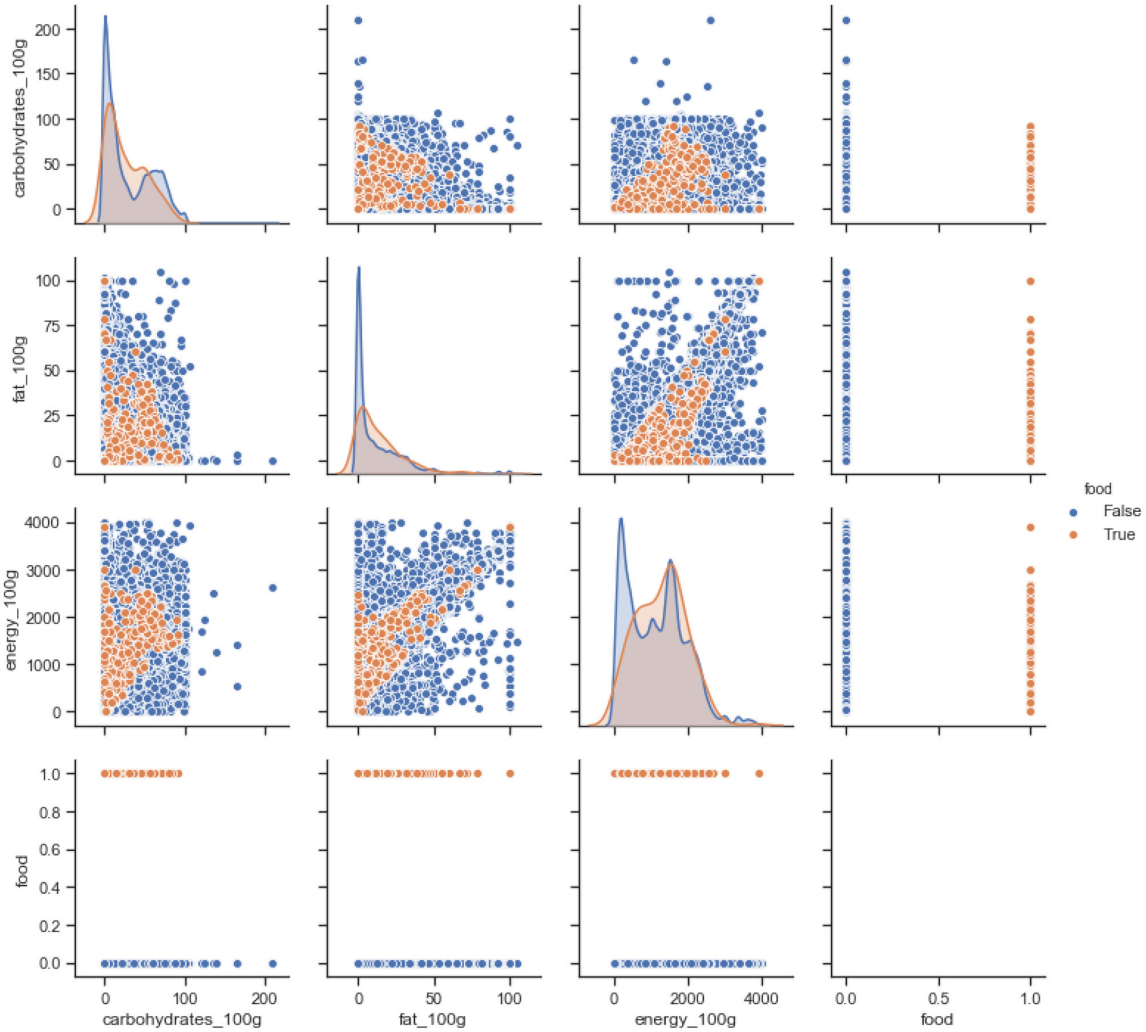
Nutrition values of vegan vs. non-vegan food in the database.

Figure 3 shows the normalized KDE graph of nutrition values of vegan and non-vegan food, where orange represents the vegan food, and blue represents the non-vegan food. The results show that the distribution is quite similar for products with “Vegan” labels. As shown in the Figure 3 non-vegan food has high fat and energy values when compared to vegan foods on average. While the scatter graph, between carbohydrates and fat, shows a vegan diet has a lower energy value when compared to non-vegan foods with regards to the same amount of carbohydrate. The last raw scatter graph shows that all our food is categorized as vegan or non-vegan food.

To differentiate between vegan and non-vegan food, our methodology involved first finding out if the word “vegan” is present beside the name of the food in the database as shown in Algorithm 1. Then vegetables, fruits, and juice were added as well to the vegan food category. Any other foods are considered as non-vegan food.

**Algorithm 1:**
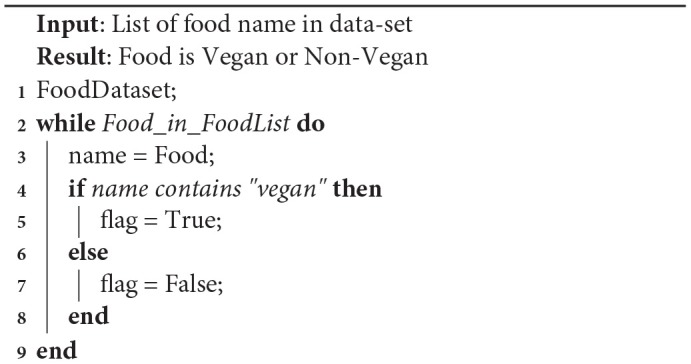
Identifying food is vegan or non-vegan

**Figure 4:**
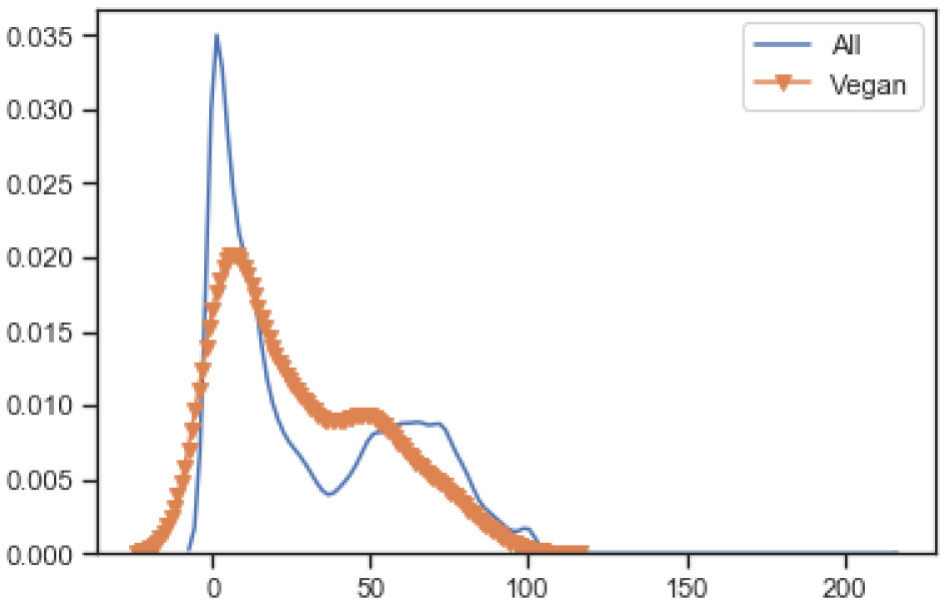
KDE of carbohydrates per 100 g.

Figure 4 shows the KDE graph of carbohydrates per 100 g concerning the distribution between vegan and non-vegan foods. It also shows that some non-vegan food has high carbohydrate content than vegan foods. While, in other aspects of nutrition, the gap between vegan and non-vegan food is not so big.

**Figure 5:**
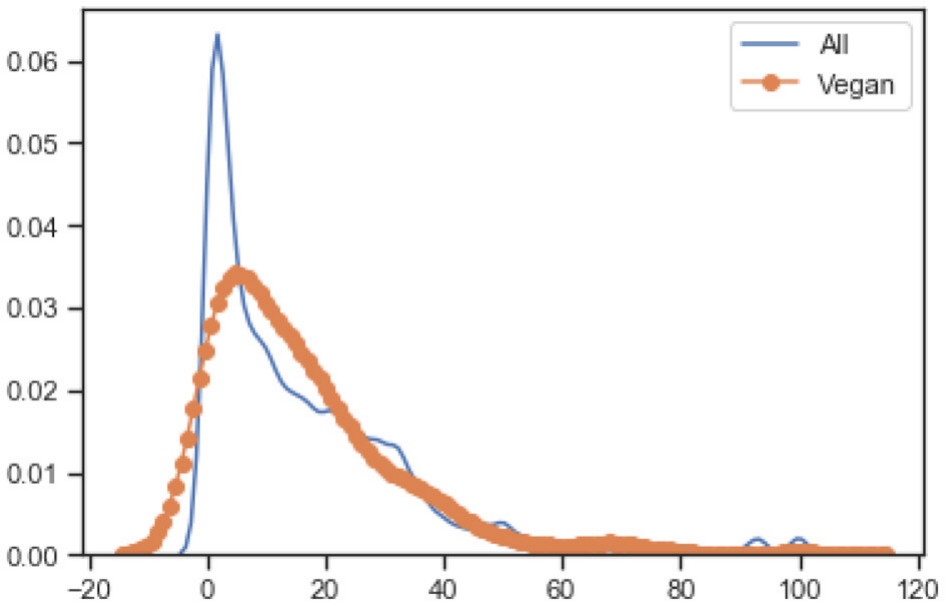
KDE of fat per 100 g.

Figure 5 shows the KDE graph of fat per 100 g distribution between vegan and non-vegan foods. It also shows that some non-vegan foods have sharply high-fat content than vegan products. In other aspects, there is not much of a big difference between vegan and non-vegan foods, for example, fat content.

**Figure 6:**
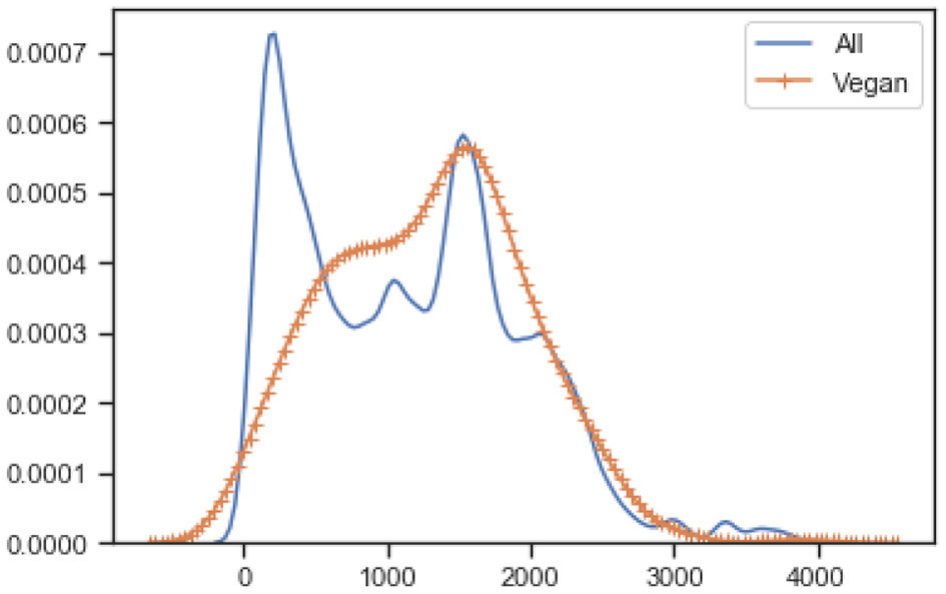
KDE of energy per 100 g.

Figure 6 shows the KDE graph of energy per 100 g distribution between vegan and non-vegan food.

In order to analyze public health, the second database needed is an activity database, where the average activity time is taken and related to its caloric values. The most common activities that are posted by people on social media were chosen. The database now contains 1, 400 different activities and their caloric values that are available. To calculate the average caloric value, fixed weight, and metabolism were used with average Canadian weight at 80.3 kg. Table 2 shows the attributes of the activity data-set.

**Table 2:** Activity database.

**Name**	**Data**
activity_name	Activity name
caloric_value	Caloric value of the activity

To analyze public health on a large scale, the Twitter data-set was considered as the primary source for data. This will be used to do basic querying and analysis of the system at a large scale; Elasticsearch based analysis system is developed for real-time querying and searching of Twitter data (17). From that system, 99, 999, 986 tweets were analyzed between 2018 and 2019.

### 3.3. Phrase Detection

In social media, a text data phrase gives more information than a single word. In previous work, one of the limitations is the inability to understand the phrases of multi-words. For example, when anyone tweets “**you are the apple of my eye**,” it considers “**apple**” as a food item. In our system apple of my eye is considered as a single phrase, which has a specific meaning. One common example as well is “**you are a smart cookie**” where “**smart cookie**” has meaning as a phrase. To overcome this limitation new features are added during the training of the NLP algorithm. This algorithm was developed initially by Williams (20), and it is based on the distance between two words. This text partitioning algorithm is based on William's fine-grained text segmentation algorithm. It considers the whole text as two parts: word and non-word tokens. The important feature of this algorithm is that it considers non-word tokens as a linker between two words. For example, in the phrase “**apple of my eye**,” “**of**,” and “**my**” are non-words and works as a joiner of the single phrase with singular meaning.

Algorithm 2 is a phrase detection algorithm that uses a concatenation operation. This links the tokens together to create forms and then finds out how related the form is to the lexicon. If the form is not correlated with the lexicon then the next possible form is analyzed. If the form is related to the lexicon, it is considered as a phrase.

**Algorithm 2:**
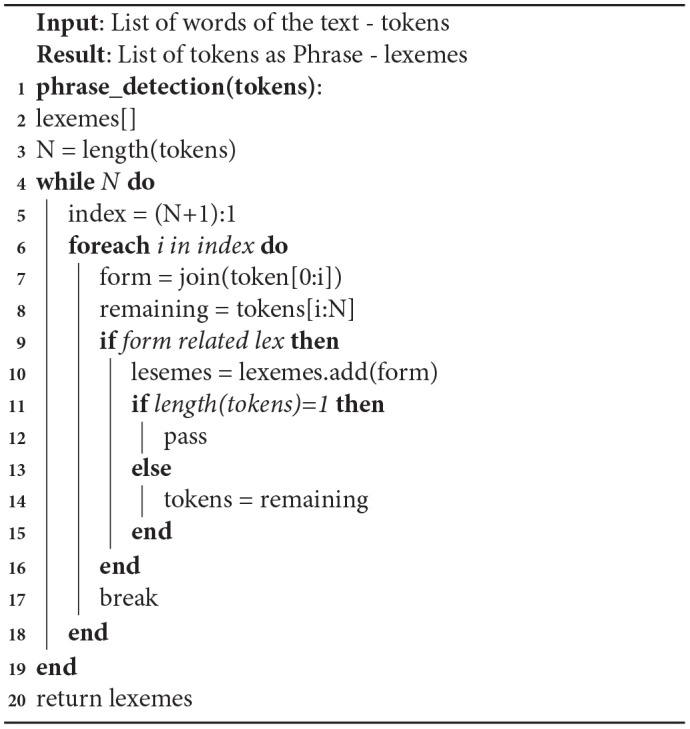
Phrase detection algorithm

Algorithm 2 is based on Boundary-based multi-word expression segmentation with text partitioning by Williams (20). This algorithm focuses on the next possible word pair, which means a lower precision and efficiency for complex bound phrases. But the phrase information will be derived from a gold standard data-set. For example, Supersense-tagged Repository of English with Unified Semantic and Riter and Lowlands data-set of superscience-annotated tweets for the SemEval in Williams (20). Due to that pre-information of the phrases finding, results with simple and common phrases are easy. Box 2 shows the results of the phrase extraction algorithm used.

Box 2 **Phase extraction results.**

text: “I saw the sweet potatoes.”

phrase: “['sweet', 'potatoes']”

text: “My daughter is an apple of my eyes.”

phrase: “['apple', 'eyes', 'daughter']”

Our results shows that the phrase detection algorithm will analyze the text and predict phrases for example the phrases “**sweet potatoes**” and “**apple eyes daughter**” are a single phrase. Even though our data cleaning process removes stop words the phrase detection algorithm can still detect a complicated sentence like “**my daughter is the apple of my eye**” as the phrase “**apple eyes daughter**.”

### 3.4. Model Training

**Figure 7:**
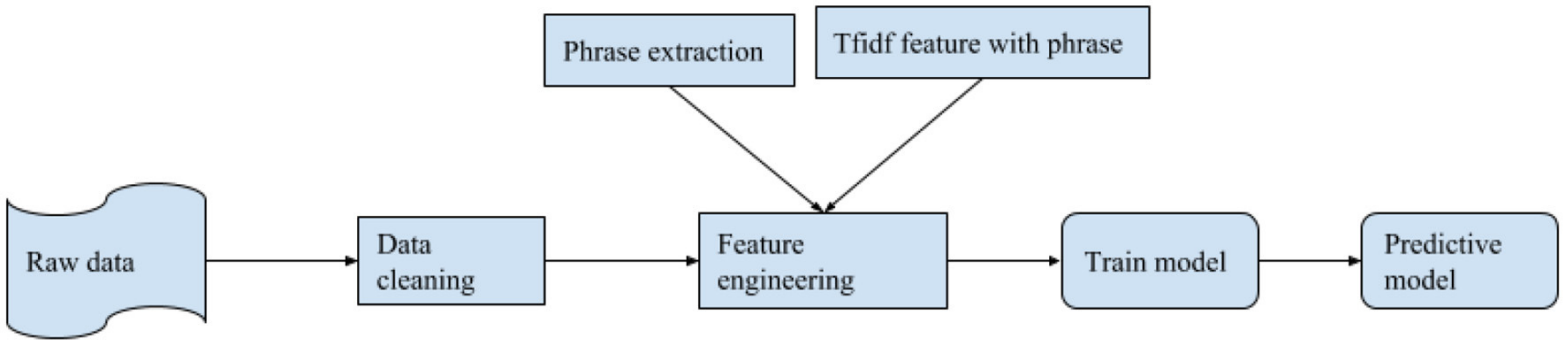
Precessing pipeline of the system.

In the next step, the machine learning model was trained for classification of the tweet. As shown in Figure 7, our first step is to take the raw data and clean it (explained in section 3). After cleaning the data, feature engineering was performed. In feature engineering, term frequency-inverse document frequency (tf-idf) is created and phrase extraction occurs. Here, phrases as a single word are used as a feature in tf-idf. After that, the model was trained and then used as a pre-trained model for binary classification of the tweet for food and non-food related tweets.

Two types of features were used: tf-idf with phrases and word embedding with 2 g. Tf-idf is used for Naïve Bayes (NB), Logistic Regression (LR), and Random Forest (RF), and Support Vector Machine (SVM). Embedding for Shallow neural network (SNN), Convolutional neural network (CNN), and Reinforcement neural network (RNN-GRU) was used.

## 4. Analysis and Results

In previous works, researchers tried to find the caloric value through non-NLP or basic NLP algorithms. Because of that, the false positive rate of data was high, and this decreased the accuracy of the results. False positive errors will start to increase as the data amounts increase (21). This affects the accuracy and accountability of the system. Many models are available for the classification of text. In this paper the binary form of classification is much easier than multi-class classification. It also removes the necessity of the necessity to use advanced deep learning algorithms. The first algorithm tested was LR. This measures the relationship between one or more categorical dependent and independent variables. It will be estimated through logistic (sigmoid is more common presently) function.

**Figure 8:**
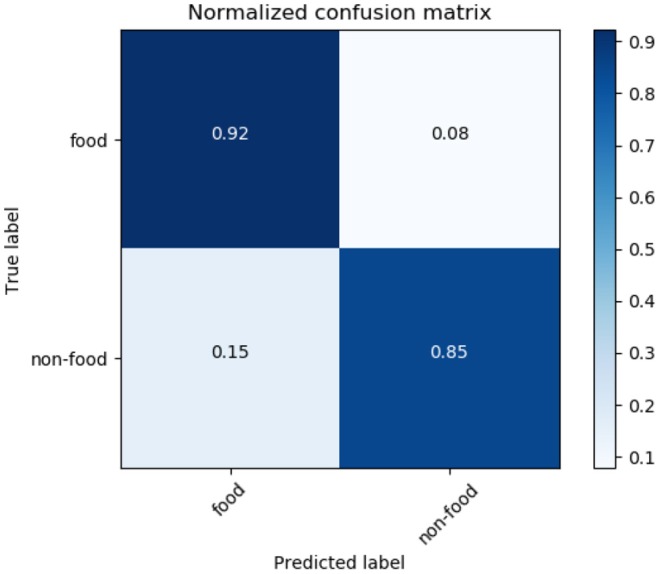
Logistic regression confusion matrix.

Figure 8 shows the confusion matrix of LR. It also shows that 92% of food tweets were successfully identified. While recognizing only 85% of the non-food tweet as non-food. But the false positive ratio is very high, 15% which landed into more noisy data.

**Figure 9:**
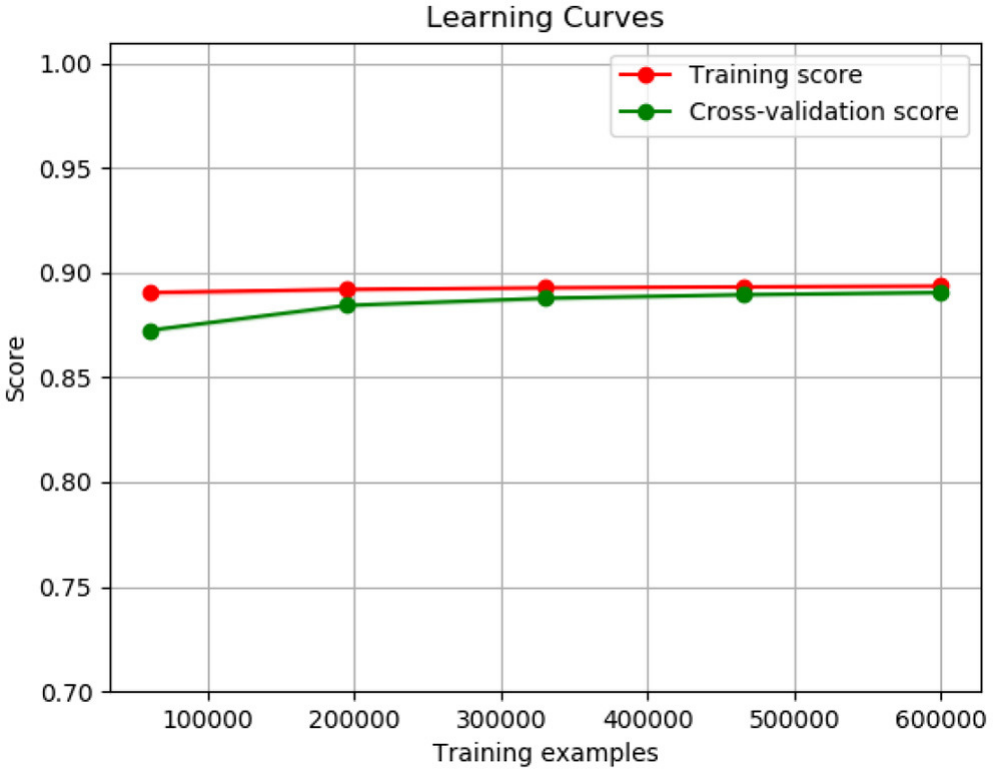
Logistic regression training curve.

Figure 9 shows the training curve of LR. As shown, as the number of training samples increases its accuracy is also increasing.

Our second algorithm is the NB algorithm with tf-idf features on a word level. This classification of algorithm techniques is based on Bayes' theorem. This assumes Independence between predictors. Meaning, it implies that one feature in the model is unrelated to another feature.

**Figure 10:**
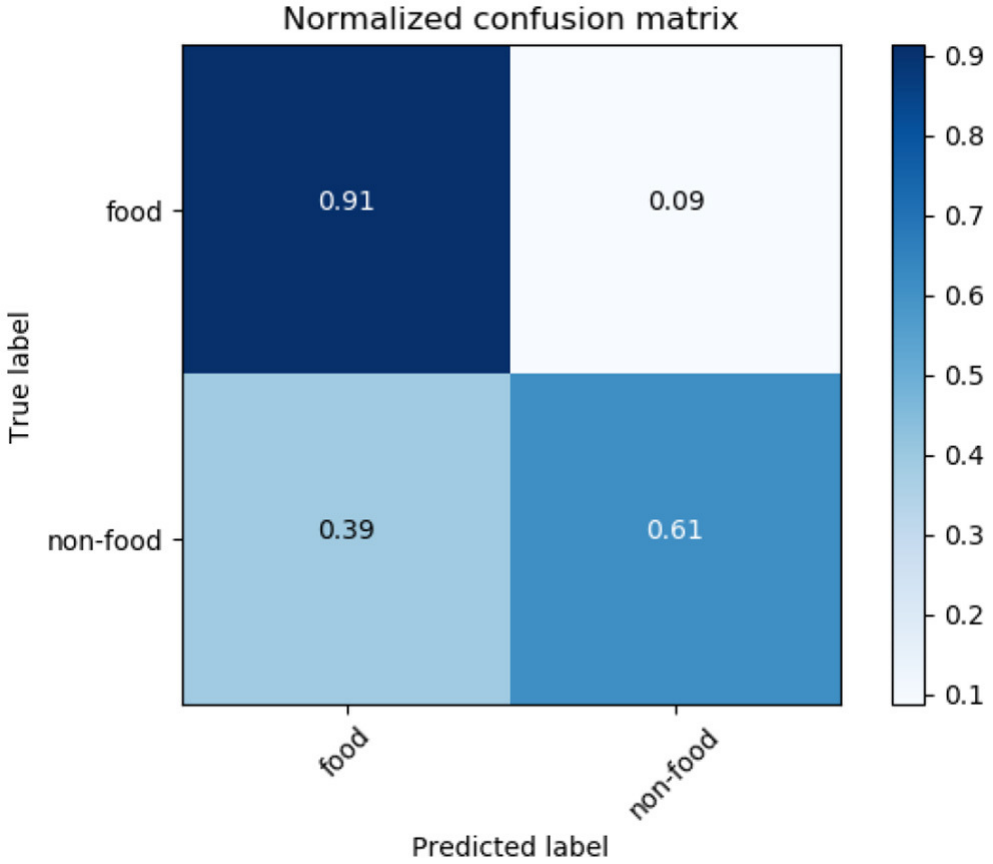
Naïve Bayes confusion matrix.

**Figure 11:**
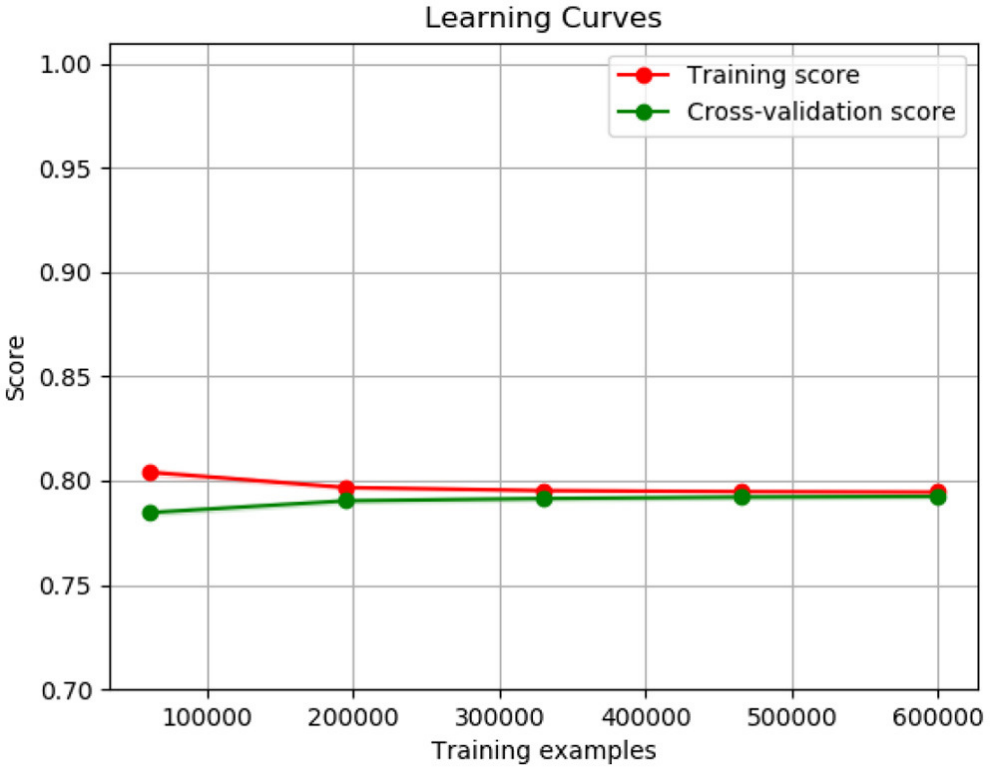
Naïve Bayes training curve.

Figure 10 shows the confusion matrix of NB. It also shows that the methodology can successfully identify 91% of food tweets as food tweet, while only 61% of the non-food tweets as non-food tweets and a high rate of false positive as 39%, which is quite high to get accurate results. Figure 11 shows the training curve of NB. It shows the accuracy of the algorithm increases as the sample size is increased. After 400 K samples, the accuracy of the algorithm is almost at 80%.

**Figure 12:**
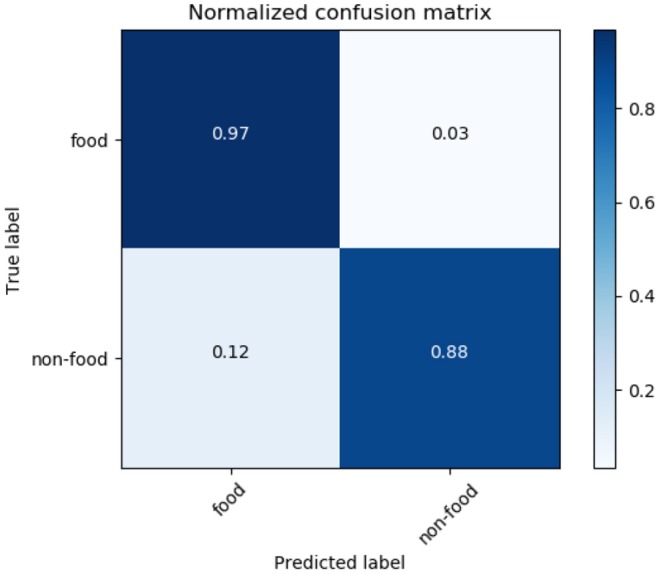
Random Forest confusion matrix.

**Figure 13:**
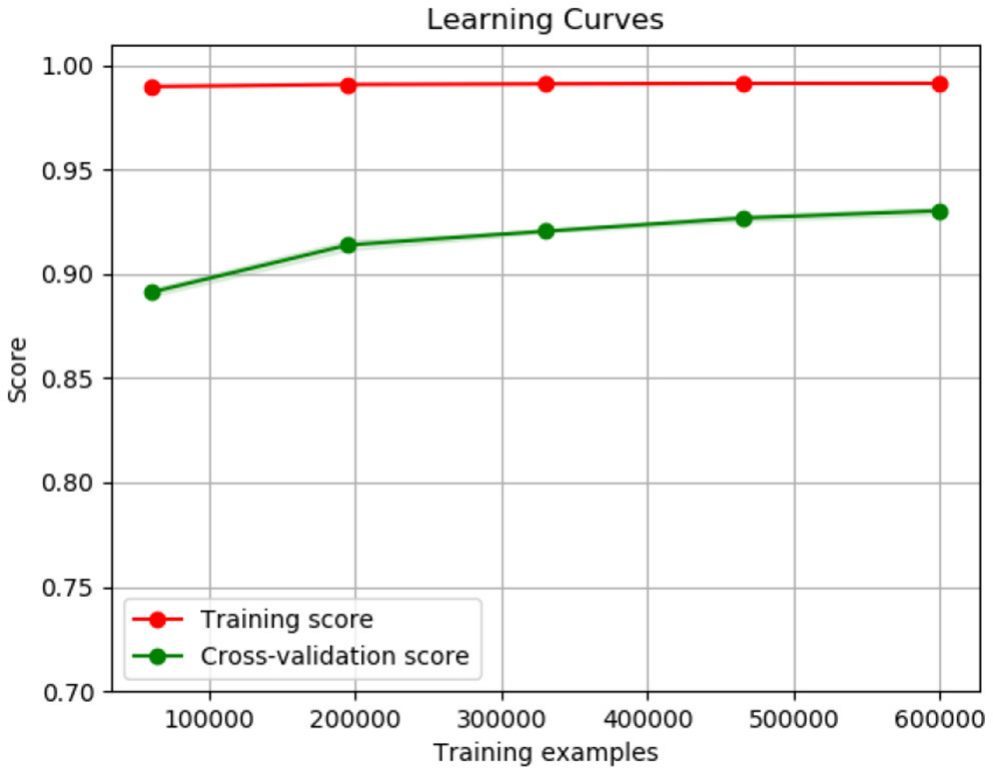
Random Forest training curve.

The third model analyzed was the RF model. This is a type of bagging model, and it is a part of the tree based model. An advantage of this model is that it gives more accurate pr editions when comparing it to any simple CART or regression model in specific scenarios. Figure 12 shows the confusion metric of RF. It also shows that our methodology successfully identified 97% of the food tweets as food tweets, while 88% of the non-food tweets are recognized as non-food tweets. On the other hand, the false positive rate is also as low at 12%. This result shows the highest accuracy among the other tested models. Figure 13 shows the learning curve of RF. It also shows that the accuracy of the algorithm increases as the data size increases.

Table 3 shows an overview of the accuracy of the different models used to test our methodology. From the above analysis, the RF model gives us the best results for the binary classification of tweets in food and non-food categories. The next step is to get information about the calories and the user's activity based on our data-set. Three different values became our focus: caloric value they gain from their food (*C*_*in*_), the caloric value they burn from their activity (*C*_*out*_), and their caloric ratio from the first two values (*C*_*rat*_) (Equation 1). Those three values are co-related to the 37 measures of the well being and health. In LCM, the authors found a statistically strong correlation between high blood pressure, inactivity, diabetics, and obesity rates (12). Now to count these three values *C*_*in*_, *C*_*rat*_, *C*_*out*_ our methodology depends on the benefits from each individual tweet.

**Table 3:** Model accuracy.

**Model**	**Accuracy %**
Naïve Bayes	79.202
Linear regression	89.155
Random Forest	93.406
CNN	60.142
RNN-GRU	60.034
SVM	56.031

**Algorithm 3:**
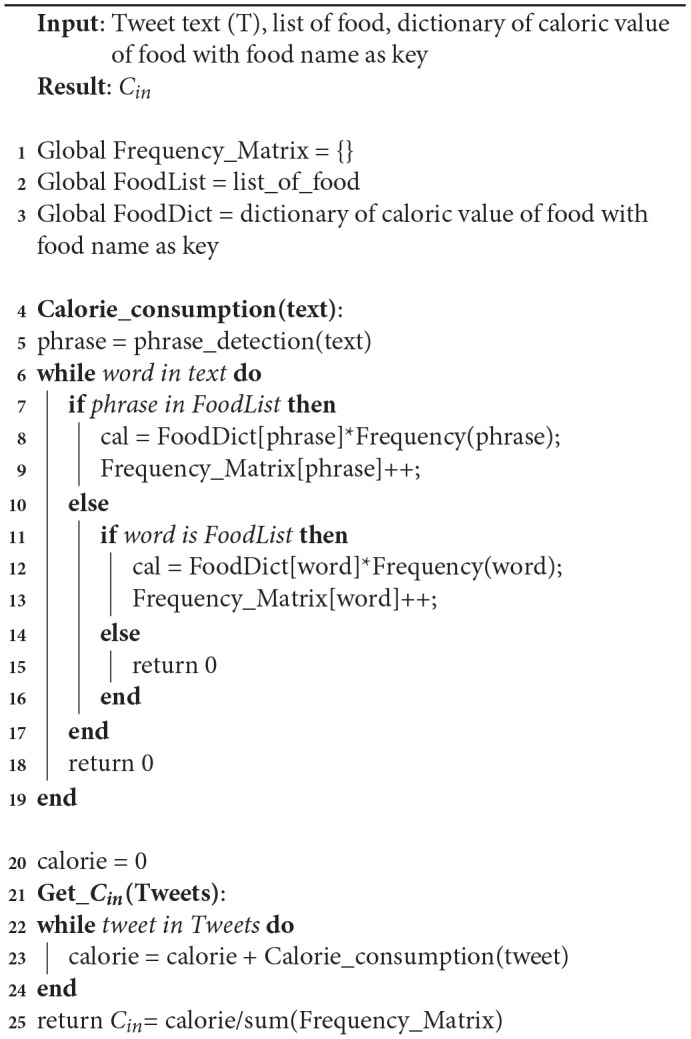
*C*_*in*_ algorithm

Algorithm 3 gives the value of *C*_*in*_ value for *C*_*rat*_ as shown in Equation (1). In the first step, tweets in our data-set are taken to produce the caloric value of each tweet using the Calorie_consumption function. In that function, text is used to locate any food phrases. In the second step, the individual words or phrases that are found in the tweet are compared to the food data-set. If present, caloric value is taken of the word or phrase from the food dictionary. Then calories of the given food are multiplied with the frequency of that word in the text. It is then stored in the Frequency matrix and to get a normalized *C*_*in*_ value. The sum of all calorie values from all the tweets are divided by the sum of the frequency matrix.

**Algorithm 4:**
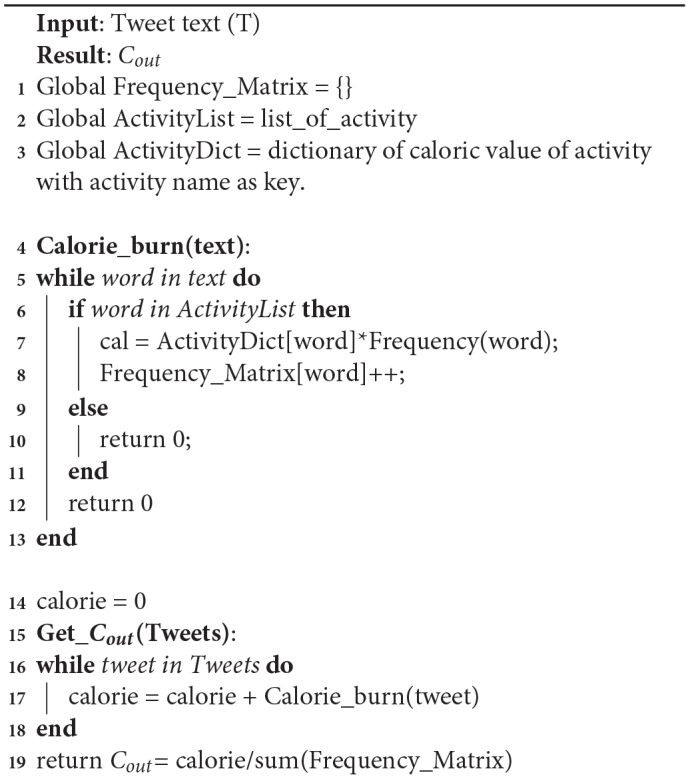
*C*_*out*_ algorithm

Algorithm 4 gives the value of *C*_*out*_ value for *C*_*rat*_ as shown in Equation (1). In the first step, tweets in our data-set are taken to get the caloric values of each possible word in the tweet. Next, each word from each tweet is checked with the activity data-set. The data-set will give a caloric burn value for each tweet. To normalize the *C*_*out*_ value, the summation of caloric values is divided by the frequency of each activity phrase. To count the *C*_*out*_ values, how many calories a person can burn is stored from a particular activity. For that, a body weight of 80.7 kg is assumed as the standard average weight of a Canadian adult.

Table 4 is the result of the 100 K tweets gathered between 2018 and 2019. 50 K tweets were chosen pertaining to food and 50 K tweets pertaining to activity from each province and territory randomly, which combine to form our 100 K tweet data-set (22). The Twitter API was used to collect the data without any filters and therefore makes our collection of tweets random (23). Table 4 shows the top 10 foods in Canada, and it clearly shows that junk food and hot drinks are the most common.

**Table 4:** Top 10 food in Canada.

**Rank**	**Food**	**Number of tweets**
1	Coffee	38,785
2	Burger	35,166
3	Pizza	34,369
4	Noodles	27,891
5	Cake	18,456
6	Pie	17,982
7	Juice	16,711
8	Tea	16,631
9	Fruits	15,987
10	Veggies	11,473

Table 5 is the list of the top 10 activities in Canadian tweets. It clearly shows that large number of people are choosing to watch something regularly instead of physical exercise. It also shows that walking and running are the most common exercises people do. This means physical inactivity is increasing throughout Canada.

**Table 5:** Top 10 activity in Canada.

**Rank**	**Activity**	**Number of tweets**
1	Watching (seeing)	42,489
2	Reading	31,762
3	Walking	28,127
4	Running	27,838
5	Drinking	27,339
6	Sitting	24,347
7	Cooking	22,561
8	Skiing	18,947
9	Gym	16,585
10	Playing	14,191

**Figure 14:**
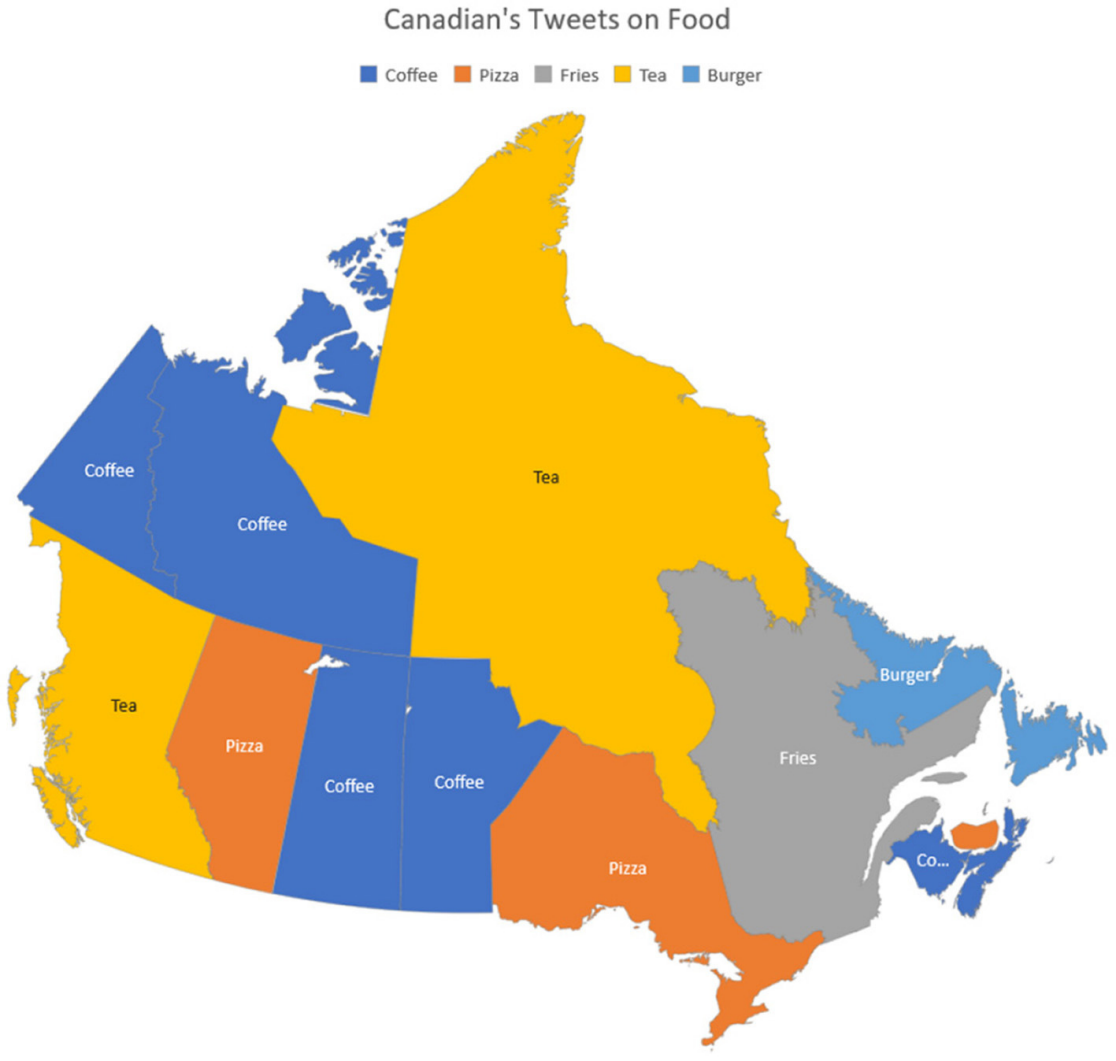
Canadian's tweets on food.

Figure 14 shows the most common foods people tweeted about in different provinces and territories of Canada. Clearly in Ontario and Alberta, the most common foods tweeted are Pizza, while Quebec's most common food is Fries. It also shows that coffee is the most common tweeted drink in provinces like Manitoba, Saskatchewan, Yukon, and Northwest Territories as compared to tea, which is more common in tweets originating from British Columbia.

**Figure 15:**
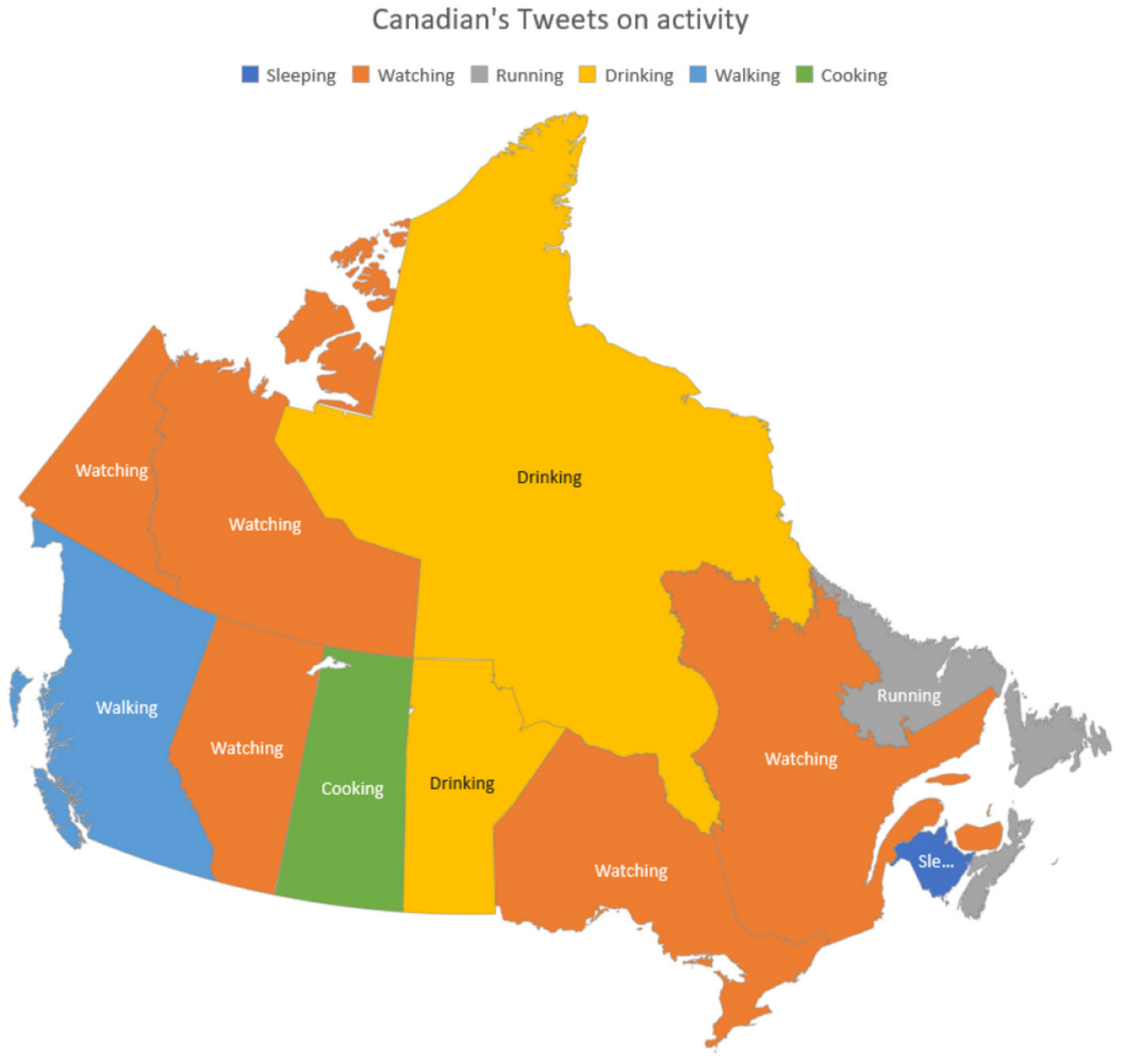
Canadian's tweets on activity.

Figure 15 is about the most common activities people tweeted about in different provinces and territories of Canada. The result shows that watching (TV) is the most common activity in dense populated provinces of Canada. This includes Ontario, Quebec, Alberta, Yukon, and Northwest Territories. This shows that there is less physical activity among people in these provinces, which is an alarming situation when looking at the individual's food consumption versus activity they do to burn calories.

From the Canadian government's own health analysis (24), it shows that Ontario and Quebec have 38.3 and 23.2% of the total population respectively. When both are combined, it is 61.5% of the total population. Caloric ratio is highly correlated to blood pressure and obesity, which according to LCM that 77.92% population has a higher chance of getting obese and/or higher blood pressure which is a staggering percentage. Our results here correlate directly to the results given by the Canadian Institute of Health Information report “**Obesity in Canada**” (25). It also shows the rapid growth of Obesity in Ontario and Quebec.

**Figure 16:**
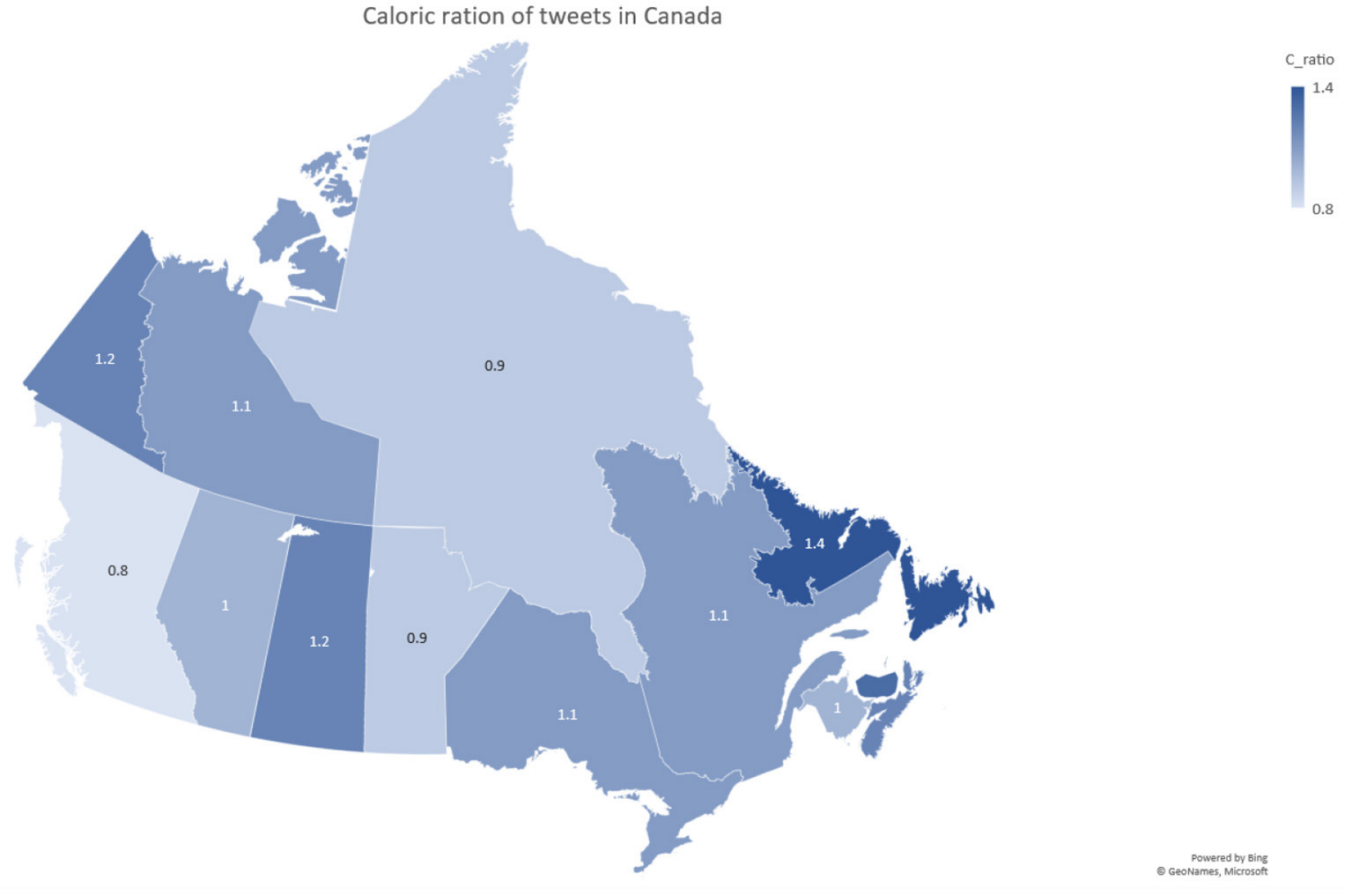
Caloric ratio of tweets in Canada.

Figure 16 shows the Caloric ratio based on Equation (1). If the ratio is >1 that means that the province's consumption is greater than the caloric usage. The opposite is true when the ratio is <1. According to Figure 16, Yukon, Newfoundland and Labrador, and Saskatchewan's caloric consumption is higher than their caloric burning at this instance. Northwest Territories, Manitoba, and British Columbia have a caloric burn that is higher than their caloric consumption. Caloric ratio is highly correlated to blood pressure and obesity (12). When looking at Figure 16, 77.92% population has a caloric ration >1.0. This can cause greater chance of getting obese, and higher blood pressure. This population estimation is based on the population numbers from the 2016 Canadian Census and was calculated by adding up the populations from each individual province with a 1.0 caloric ratio or higher. This is alarming because it represents such a huge part of Canada's population.

Obesity percentages for the provinces of Canada are analyzed in Figure 16. When looking at the Obesity in Canada report, published in 2017, the order of provinces from lowest obesity rate to highest obesity rate is the following:

1. British Columbia
2. Quebec
3. Ontario
4. Alberta
5. Manitoba
6. Saskatchewan
7. Newfoundland and Labrador (25).

When comparing that with the lowest to highest rank in Figure 16, British Columbia is clearly the lowest, while Quebec and Ontario are tied, and Alberta and Manitoba are switched in ranks, Saskatchewan and Newfoundland and Labrador are the highest. The significance of the comparison above is that it shows a strong correlation between Figure 16 and the data from the report. The provinces with the highest ratios also have the highest obesity rates and the opposite is also true. This goes as far as to show that our data can be just as reliable as a published Canadian report. By using this knowledge, the Canadian government can target healthy living programs in the provinces that need it like Newfoundland and Labrador.

Figure 16 only shows one caloric ration per province from the tweets collected at that instance. In order to become obese you need to constantly have a ratio >1 over the span of weeks. That way you are consuming more calories that you are burning which leads to the gaining of weight. Therefore, our Figure 16 can be considered as one data point in an obesity rate trend and in the future it can be added with many other points taken in different times to accurately show the rate of obesity in Canada. By this logic you can also see the trend from one point to another and immediately see if the programs implemented to counteract obesity have worked. The result is going from tweets to a real time analysis of the rate of obesity in Canada.

## 5. Conclusion and Future Work

Developing high-performance machine learning model with a limited amount of training data is always a challenge, as it restricts the use of more complex deep learning and neural models. Our model gives 93.406% accuracy in binary classification of food and non-food tweet. This result shows that social media analysis on a large scale with the use of better NLP algorithms can help us to identify food and activity related tweets more accurately. This helps us to gain a larger perspective on daily activities and its effect on people's health. Our results convey a complex relationship between health and social media. The presented approach is faster when compared to traditional survey methods causing data to be readily available as well a close representation of real time. Here, many promising future works are possible such as a more dynamic way to calculate calories based on age, gender and work profile. Another limitation is that when our model looks at the tweet it only recognizes the food but it leaves out the quantity of said food. In example, our model will not be able to differentiate 1 apple from 10 apples.

## Data Availability Statement

Publicly available datasets were analyzed in this study. Authors interested in accessing the data can contact us at: https://www.datalab.science/.

## Author Contributions

NS developed the algorithm and wrote the first draft. GS and DS provided guidance to conduct the research. VM supervised the research and helped in all aspects of this research.

### Conflict of Interest

The authors declare that the research was conducted in the absence of any commercial or financial relationships that could be construed as a potential conflict of interest.
